# Caregiving and Place: Combining Geographic Information System (GIS) and Survey Methods to Examine Neighborhood Context and Caregiver Outcomes

**DOI:** 10.1093/geroni/igz025

**Published:** 2019-08-23

**Authors:** Scott R Beach, Ellen Kinnee, Richard Schulz

**Affiliations:** University Center for Social and Urban Research, University of Pittsburgh, Pennsylvania

**Keywords:** Caregiving informal, Environment, Quantitative research methods

## Abstract

**Background:**

Little is known about the impact of neighborhood context on family caregivers, or how environmental factors combine with individual-level caregiver risk factors to affect caregiver outcomes.

**Objectives:**

To combine Geographic Information System (GIS) and survey methods to examine the effects of caregiver residence in disadvantaged/underserved neighborhoods on caregiver outcomes.

**Research Design and Methods:**

Telephone surveys with 758 caregivers from the Pittsburgh Regional Caregiver Survey geocoded for classification into Environmental Justice Areas (EJAs) and Medically Underserved Areas (MUAs). We examine the impact of EJA/MUA caregiver residence on care recipient unmet needs for care, caregiver depression and burden, and positive aspects of caregiving, adjusting for sociodemographics, caregiving context, care recipient disability level, caregiving intensity, and additional risk factors.

**Results:**

There was spatial clustering of caregiver depression and burden outside of the disadvantaged/underserved areas, while positive aspects of caregiving were clustered within EJAs/MUAs. Approximately 36% of caregivers lived in EJAs/MUAs, and they differed, sociodemographically, on caregiver risk factors and caregiver outcomes. Multivariable models showed that caregivers residing in EJAs/MUAs were less likely to be depressed and reported more positive aspects of caregiving after adjusting for known individual-level risk factors. Residence in disadvantaged/underserved areas also modified the effects of several risk factors on caregiver outcomes.

**Discussion and Implications:**

Caregiver outcomes show interesting spatial patterns. Unexpectedly, caregivers living in these potentially challenging environments were less depressed and reported more gains from caregiving after adjusting for known risk factors. Results suggest that socioeconomic disadvantage does not necessarily translate into poor caregiver outcomes. Understanding the mechanism for these effects is important to designing effective caregiver interventions. The paper also demonstrates the value of using GIS methods to study caregiving.

Translational Significance Results suggest that socioeconomic disadvantage does not necessarily translate into poor caregiver outcomes and, in fact, is associated with lower caregiver depression and more positive aspects of caregiving. Understanding the mechanism for these effects is important to designing effective caregiver interventions. Interventions for caregivers in disadvantaged areas could build upon psychological strengths and resources, while those for caregivers in other areas should focus on enhancing mental health.

There is substantial and growing interest in “aging in place,” seen as a desirable, cost-effective alternative to formal long-term care for older adults. Unpaid family caregivers play a crucial role in allowing disabled older adults to remain in their homes (Schulz & Eden, 2016). While empirical research on neighborhood contextual factors and health outcomes of older adults has been increasing (Pruchno, 2018), relatively little attention has been given to environmental context in the caregiving literature. For example, evidence suggests that the neighborhood environment may serve to modify the effects of personal stress on health outcomes for older adults more generally (Ferris, Glicksman, & Kleban, 2016; Siegler, Brummett, Williams, Haney, & Dilworth-Anderson, 2010; Stahl, Beach, Musa, & Schulz, 2017). What is not known is how the neighborhood, where a caregiver lives, affects his/her ability to provide quality care to a loved one. Also, what effect does environmental context have on adverse caregiver outcomes like burden and depression, or the ability to derive benefits from the caregiving experience, which is a growing area of focus in the literature (R. M. Brown & S. L. Brown, 2014; Roth, Fredman, & Haley, 2015)? The answers to these questions have important implications for identifying caregivers at risk for adverse outcomes and the design of interventions to support them.

Decades of research have revealed numerous risk factors for adverse outcomes due to family caregiving (Adelman, Tmanova, Delgado, Dion, & Lachs, 2014; Pinquart & Sörensen, 2003; Schulz & Eden, 2016). These include sociodemographic factors (low income and education, older age, spouse caregiving, co-residing with the recipient); intensity of caregiving (high recipient disability levels, >100 hr of care provided per month, dementia care); caregiver perceptions of the recipients suffering; lack of choice in becoming a caregiver; poor caregiver physical health; and lack of social and professional supports. This paper explores how potentially disadvantaged caregiver neighborhoods may add to or modify known risk factors to affect care recipient unmet needs for care, caregiver depression, caregiver burden, and positive aspects of caregiving. We use Geographic Information System (GIS) methods to geocode and classify caregiver neighborhoods as potentially disadvantaged (using recognized Environmental Justice Area [EJA] and Medically Underserved Area [MUA] designations, described below) with linkage to caregiver survey responses to explore the role of “place” in family caregiving. We also examine this relative disadvantage in the context of urban versus non-urban caregiver neighborhoods.

GIS and geocoding, the process of assigning x and y coordinates (latitude and longitude) to address data, are commonly employed methods that allow for characterization of residential neighborhood environments in health-related research, but they have rarely been used to examine caregiver outcomes. Some differences in outcomes have been reported between family caregivers living in rural and urban areas, which have been related to resource gaps, lack of public transportation, and access to healthcare providers (Crouch, Probst, & Bennett, 2017), but these studies have not used GIS methods.

A few studies have used GIS to examine the effect of neighborhood environments on older adults in general. A census block-level analysis in Ohio found limited access to healthy foods among older people with transportation or mobility limitations (Yamashita & Kunkel, 2012). Age-stratified vulnerability scores developed in GIS were used to analyze patterns of socially and medically vulnerable older adults in southern Florida (Hames, Stoler, Emrich, Tewary, & Pandya, 2017). Key neighborhood factors contributing to social vulnerability included race and income. Medical vulnerability was driven by disease burden and access to emergency cardiac services. Only two studies, we are aware of, used GIS in the context of family caregiver outcomes. GIS analyses in Ireland examined locations of unpaid care providers, the disabled, and the elderly to identify areas of caregiving deficits (Kalogirou & Foley, 2007). One U.S.-based GIS study (Brummett et al., 2005) found that effects of caregiving on glucose levels are magnified by negative neighborhood characteristics. Impaired glucose metabolism is strongly associated with increased stress and may serve as a link to cardiovascular disease.

There are a number of demographic and economic factors that can be used to characterize neighborhoods, and we focused on two types of areas with particular relevance to caregiving. EJAs are defined by the Pennsylvania Department of Environmental Protection (PADEP) as any census tract where at least 20% of the population lives in poverty and/or 30% or more of the population is minority (PADEP, www.dep.pa.gov). These areas represent disadvantaged communities which often face greater likelihood of exposure to air pollution (Hajat, Hsia, & O’Neill, 2015; Hill, Jorgenson, Ore, Balistreri, & Clark, 2019), unequal access to healthy food (A. Hilmers, D. C. Hilmers, & Dave, 2012), and experience socioeconomic factors which may modify health effects by influencing individual vulnerability (White, Haas, & Williams, 2012). MUAs, generated by the U.S. Health Resources and Services Administration, are defined by using the Index of Medical Underservice (IMU) which is based on four variables: (1) ratio of primary care physicians to 1,000 population, (2) percentage of the population below the federal poverty level, (3) percentage of the population age 65 and older, and (4) infant mortality rate (HRSA, data.hrsa.gov). The MUA designation directly incorporates both primary care accessibility and need-related variables and serves as a measure of health care shortage. These areas show lower cancer screening rates (Wong, 2015) and increased cardiovascular risk factors (Allen et al., 2011). Medicare beneficiaries in these areas have been found more likely to experience potentially preventable hospitalizations (Parchman & Culler, 1999).

Both areas are generated using Census Tract-level data; however, EJAs are defined solely by race and low income, while the MUA criteria also include reduced access to primary health care. Research has shown that improved primary care utilization is associated with better health outcomes for Medicare recipients (Ferrante et al., 2013; Macinko, Starfield, & Shi, 2007) mostly due to increased health care utilization including screening for common ailments. In this study, EJAs and MUAs are used to represent the potentially increased stress associated with living in an economic and socially disadvantaged neighborhood environment. Stressors act on health through multiple physiological (e.g., regulation of stress response) and psychological (e.g., threat appraisal, social cognition) responses (American Psychological Association, 2017). On this basis, neighborhood context may contribute to an understanding of the role of socioeconomic stress and its contribution to caregiver health outcomes.

In this paper, we integrate GIS methods with a caregiver survey that comprehensively measures individual-level risk factors for adverse caregiver outcomes. The paper combines the survey and GIS data to answer the following research questions:

1. How many caregivers live in potentially disadvantaged areas or MUAs, and how do they differ from non-disadvantaged caregivers in terms of sociodemographics, caregiving context, care recipient disability level, caregiving intensity, additional caregiver risk factors, and caregiver outcome measures?
2. Does living in a disadvantaged/underserved neighborhood have a direct or main effect on caregiver outcomes once individual-level risk factors have been accounted for?
3. Do the effects of the individual-level risk factors vary by or depend on whether the caregiver lives in a disadvantaged/underserved neighborhood? That is, does the neighborhood serve as an effect modifier or moderator of the effects of individual-level risk factors on caregiver outcomes?

Given the sparse literature on neighborhood effects on caregiving, we make no formal specific hypotheses. However, we speculated that living in a disadvantaged/underserved neighborhood might serve as an additional stressor and thus be independently associated with negative caregiver outcomes after adjusting for individual-level risk. In addition, we reasoned that living in a disadvantaged/underserved neighborhood would magnify or intensify the negative impact of individual-level risk factors on caregiver outcomes.

## Design and Methods

### Participants

Participants were from the 2017 Pittsburgh Regional Caregivers Survey, which involved telephone interviews with 1,008 caregivers. Caregivers were unpaid friends and relatives taking care of adults aged 50 years and older living in Pittsburgh, Pennsylvania and vicinity. The caregivers could be of any age (18 and older). This study focused on the subset of 792 caregivers in Allegheny County (which includes Pittsburgh), as GIS and geocoding using our methods (see below) are less reliable in less urban areas.

### Sampling and Data Collection

A variety of sampling and recruitment methods were utilized to identify caregivers, including random digit dialing (RDD) of landline and cellular phones, listed household samples, research registries, and recruitment flyers through local service providers. Caregivers were screened and included in the study if they provided care to a relative, partner, or friend aged 50 years or older. The following questions were used to screen for caregivers: (1) “Are you currently providing unpaid care to a relative, partner, or friend aged 50 years or older to help them take care of themselves because of a chronic illness or disability? This may include helping with personal needs, household chores, or medical/nursing tasks. It might also be managing a person’s finances or arranging for outside services. This adult need not live with you.” To be eligible, respondents had to answer “yes” to item 1, AND report helping with (2) personal care, (3) household, or (4) medical/nursing tasks (“yes” to at least one of the three).

Telephone interviews were conducted by staff from the University Center for Social and Urban Research (UCSUR) at the University of Pittsburgh from February 2017 to July 2017. The survey took approximately 60 min and was approved by the Institutional Review Board. Respondents received a $15 debit card for participating. The cooperation rate was 67.8% among identified eligible caregivers.

### GIS Methods

GIS and geocoding, the process of assigning x and y coordinates (latitude and longitude) to address data, are commonly employed methods that allow for characterization of neighborhood environments. Caregivers were asked to provide the name of their street and the nearest cross-street (i.e., intersection), thus avoiding asking for an exact address. Of the original 792 Allegheny County-residing caregivers surveyed, 779 (98.3%) provided street and cross-street information. Caregiver cross-streets were geocoded using the ArcGIS World Geocoding Service (ESRI, 2018). A total of 758 of the available 779 caregivers (97.3%; 95.7% of the total) were successfully geocoded. In sum, only 1.7% of the sample refused to provide street and cross-street data; and an additional 2.6% provided inaccurate data that could not be geocoded.

EJA polygon features were downloaded from the Western Pennsylvania Regional Data Center (WPRDC) at UCSUR (WPRDC, wprdc.org). MUAs are generated by the U.S. Health Resources and Services Administration which identifies them as geographic areas with a lack of access to primary care medical services. Urbanization area classifications were obtained from ArcGIS Business Analyst (ESRI, 2017).

The majority of the sample lived in areas classified as neither EJA nor MUA (*n* = 470; 62.1%). Caregivers residing in either an EJA or an MUA (*n* = 159; 20.9%) and those residing in areas classified as both EJA and MUA (*n* = 129; 17.0%) were compared. The 758 caregivers were also classified as urban (*n* = 370) or semi-rural (*n* = 365) or rural (*n* = 23); and the analyses were done comparing urban (370) and non-urban (388) caregivers.

### Survey Measures

Survey measures used in these analyses consisted of six broad categories: (1) caregiver and care recipient sociodemographics, (2) caregiving context, (3) care recipient disability level, (4) caregiving intensity, (5) additional caregiver risk factors, and (6) caregiver outcome measures.

#### Caregiver outcome measures

There were four caregiver outcomes: (1) care recipient unmet needs for ADL/instrumental ADL (IADL)/mobility assistance, (2) caregiver depression, (3) caregiver burden, and (4) positive aspects of caregiving. Unmet care recipient needs were measured by asking caregivers a series of questions (for each of 11 tasks of daily living) to determine how often the care recipient needed help with the task during the past month (every day, most days, some days, rarely, never); how often the caregiver helped with the task in the past month (same scale); and whether any other unpaid or paid helpers assisted with the task. For each of the 11 tasks (household chores, shopping, ordering medications, bills/banking, eating, bathing, dressing, toileting, bed transfers, moving about the home, leaving the home), unmet care recipient need was defined as the caregiver providing less help than needed (e.g., caregiver helps some days when help is needed every day) AND no other unpaid or paid helper assisting. For analysis, caregivers were classified as having a care recipient with at least one unmet need versus no unmet needs. Caregiver depression was measured with the two-item version of the Patient Health Questionnaire (PHQ), and a cutoff score was used to classify caregivers as at risk for depression versus not (National Study of Caregiving [NSOC], www.nhats.org). Items asked how often during the past month that caregivers had “little interest or pleasure doing things,” and “felt down, depressed, or hopeless.” Caregiver burden was a seven-item index including how often (“very much,” “somewhat,” “not so much”) the caregiver reports being “exhausted when you go to bed at night,” “have more things to do than you can handle,” “don’t have time for yourself,” “as soon as you get a routine going, CRs needs change.” Three additional items asked if helping the recipient is financially, emotionally, and physically difficult for them (yes/no). The index (range = 0–7) consisted of a count of “very much” responses to the first four items and “yes” responses to the last three items. Positive aspects of caregiving was the sum of four items (range = 0–8): Helping him/her “has made you more confident of your abilities,” “has taught you to deal with difficult situations,” “has brought you closer to him/her,” and “gives you satisfaction that he/she is well cared for” ( “not so much” [0], “somewhat” [1], and “very much” [2]).

#### Sociodemographics

Caregiver and care recipient age and sex, caregiver race, caregiver education, and caregiver income were included (see Table 1 for coding). Given the small sample sizes in the top income category in the EJA/MUA and AJA & MUA groups, the top two categories (i.e., >$50,000) were combined for multivariable models (see below).

**Table 1.** Sociodemographic, Caregiving Context, Care Recipient Disability, and Caregiving Intensity Descriptive Statistics for the Total Sample, by Neighborhood Type and by Urban/Non-Urban Caregivers

		Neighborhood type			Urban/non-urban		
	Overall	Neither EJA nor MUA	EJA or MUA	EJA & MUA	Non-urban	Urban
	*N* = 758	*n* = 470	*n* = 159	*n* = 129	*n* = 388	*n* = 370
Age						
Mean CG age	58.4	59.8	56.2	55.8**	60.3	56.4**
Mean CR age	78.7	80.2	76.7	75.5**	79.9	77.4**
Sex						
Female CG, %	75.5	76.2	79.2	68.2	75.8	75.1
Female CR, %	64.9	63.0	67.9	68.2	64.4	65.4
Race (CG)						
Non-Hispanic white (ref), %	77.0	93.0	66.0	32.6	91.0	62.4
Non-Hispanic African-American, %	18.3	3.8	26.4	61.2	7.2	30.0
Other race (ref), %	4.6	3.2	7.5	6.2**	1.8	7.6**
Education (CG)						
High school or less (ref), %	18.5	16.6	17.7	26.4	18.4	18.6
Some college or associate degree, %	33.9	29.4	38.0	45.0	29.5	38.4
Bachelor’s degree, %	25.9	28.4	25.9	17.1	29.5	22.2
Master’s degree or higher, %	21.7	25.6	18.4	11.6**	22.5	20.8*
Income (CG)						
≤$20,000, %	18.0	12.5	23.5	31.5	10.2	26.2
$20,001–$50,000 (ref), %	31.5	29.2	29.4	42.6	30.5	32.5
$50,001–$100,000, %	32.9	34.4	40.4	17.6	37.4	28.1
>$100,000, %	17.7	23.8	6.6	8.3**	21.9	13.2**
Relationship of CG to CR						
Adult child, %	53.7	56.2	53.5	45.0	55.9	51.4
Spouse, %	20.1	22.1	18.2	14.7	24.0	15.9
Other (ref), %	26.3	21.7	28.3	40.3**	20.1	32.7**
CG–CR co-reside, %	42.1	44.3	41.5	34.9	44.8	39.2
CR lives alone, %	32.1	30.6	30.2	39.5	30.7	33.5
# other CGs						
None, %	34.2	35.1	32.1	33.3	34.5	33.8
One, %	15.2	15.1	14.5	16.3	13.7	16.8
Two or more, %	50.7	49.8	53.5	50.4	51.8	49.5
Employed, %	48.2	47.0	45.3	55.8	45.9	50.5
With children in household, %	17.9	17.0	17.6	21.7	16.5	19.5
Caring for other than CR, %	41.3	40.6	42.1	42.6	39.9	42.7
CR disability level						
Neither AD nor three or more ADLs (ref), %	54.7	55.3	51.6	56.6	54.4	55.1
AD only, %	11.5	13.0	10.1	7.8	12.9	10.0
Three or more ADLs only, %	22.0	19.4	27.7	24.8	20.9	23.2
AD and three or more ADLs, %	11.7	12.3	10.7	10.9	11.9	11.6
Duration of CG						
≤3 months, %	5.5	5.7	4.4	6.2	4.6	6.5
4–12 months, %	10.3	10.4	11.9	7.8	10.3	10.3
1–2 years, %	20.3	19.8	21.4	20.9	20.1	20.5
3–5 years, %	27.7	29.6	26.4	22.5	26.5	28.9
>5 years, %	36.1	34.5	35.8	42.6	38.4	33.8
Hours per week of caregiving (CG)						
≤8, %	42.5	41.4	42.0	46.9	42.7	42.2
9–19, %	24.7	26.1	23.6	21.1	24.2	25.2
20–39, %	17.1	16.8	19.1	15.6	16.4	17.8
≥40, %	15.8	15.7	15.3	16.4	16.7	14.8

*Note*: AD = Alzheimer’s disease; ADL = activities of daily living; CG = caregiver; CR = care recipient; EJA = Environmental Justice Area; MUA = Medically Underserved Area; ref = regression reference group.

**p ≤ .01; *p ≤ .05 for neighborhood type; urban/non-urban in ANOVA (age) or χ2 test.

#### Caregiving context

This was measured by caregiver/care recipient relationship (see Table 1): whether the caregiver and care recipient co-reside, and whether the care recipient lives alone. To capture additional caregiver roles that might impact outcomes, we also measured the number of additional unpaid caregivers helping the care recipient, whether the caregiver was employed outside the home, the presence of children under 18 in the household, and whether the caregiver was providing care for someone in addition to the care recipient.

#### Care recipient disability level

We used an existing four-level classification system to characterize severity of care recipient disability (Beach & Schulz, 2017): (1) neither needs help with three or more activities of daily living (ADL) nor has Alzheimer’s disease (AD); (2) has AD but less than three ADL needs; (3) does not have AD, but needs help with three or more ADL tasks; and (4) has both AD and needs help with three or more ADL tasks.

#### Caregiving duration and intensity

These are measured as the length of time the caregiver has been helping the care recipient, and the number of hours per week the caregiver reports helping the care recipient, as coded in Table 1.

#### Additional caregiver risk factors

These are additional factors associated with caregiver outcomes reported in prior studies (Schulz & Eden, 2016). Caregiver perceptions of care recipient suffering during the past month is the mean of two questions rating perceived physical and psychological suffering where 1 equals “has not been suffering” (psychologically/physically) and 10 is “has been suffering terribly” during the past month. Caregivers were also asked a simple yes/no question, “Do you feel you had a choice in taking on this responsibility for caring for CR?” The general self-rated health question was used as a proxy indicator for caregiver overall health, and was dichotomized into “fair/poor” versus “excellent/very good/and good” for analysis. Caregiver social support is the sum of two items: “Do you have friends or family that you talk to about important things in your life?” and “Do you have friends or family that help you with your daily activities, such as running errands, or helping you with things around the house?” The social support score is the number (0, 1, or 2) of the two questions answered “yes.”

### GIS Statistical Analysis and Mapping Methods

As noted above, caregiver cross-streets were extracted from the survey and geocoded using the ArcGIS World Geocoding Service (ESRI, 2018). Geocoded point locations were linked to the surveys in order to map responses and then spatially joined with EJAs, MUAs, and urbanization areas to classify each caregiver location. Two types of maps were generated to display spatial patterns in caregiver survey responses. The binary variables (i.e., unmet care recipient needs, caregiver depression) were mapped as point densities. Caregiver locations with a value of “1” for these variables were extracted, and the total number points that fall within a defined neighborhood around each location divided by the neighborhood area. Each result was displayed as a “heat map” showing areas with higher or lower concentrations of caregivers with unmet needs or depression. The continuous response variables (caregiver burden, positive aspects of caregiving) were mapped as spatial clusters. Clusters were generated using the Cluster and Outlier Analysis tool in ArcGIS 6.1 (ESRI, 2018). This tool calculates a local Moran’s *I* value (Anselin, 1995), a *z*-score, and a cluster type code for each statistically significant feature. Points with high or low cluster type codes are mapped to identify caregivers falling in clusters of similarly high or low values.

### Statistical Analysis of Survey and Neighborhood Data

Descriptive statistics are used to characterize the survey sample on all variables, both for the total sample and by neighborhood type—neither EJA nor MUA, EJA or MUA (“EJA/MUA”), EJA & MUA, and urban/non-urban. Bivariate comparisons for by neighborhood type and urban/non-urban status are made using χ ^2^ statistics for categorical variables and analysis of variance (ANOVA) for continuous variables. Then, multivariable logistic regression models are estimated with the dichotomous care recipient unmet needs and caregiver depression outcomes as dependent variables. Two models are estimated for each outcome: (1) an individual-level survey model, including all sociodemographic, caregiving context, care recipient disability, caregiving intensity, and additional caregiving risk factors as predictors; and (2) a model adding neighborhood-level factors as predictors. Dummy indicator variables were created for: (1) residing in either an EJA or MUA and (2) residing in area designated as both an EJA and MUA. Residing in neither an EJA nor an MUA was the reference category. A dummy indicator variable was created for residing in an urban neighborhood, with non-urban as the reference category. Given that caregiver burden is a count index variable, negative binomial models were estimated for this outcome. Ordinary least squares (OLS) regression models were run for the continuously scored positive aspects of caregiving outcome. The aim of these models was to estimate the main effects of residing in specific neighborhood types (EJA/MUA; EJA & MUA), and in urban (vs. non-urban) neighborhoods on the caregiver outcome variables. Lastly, in order to test for potential moderator effects of neighborhood, separate logistic, negative binomial, and OLS models were estimated using individual-level survey predictors and the urban indicator: (1) within the subsample living in areas defined as neither EJA nor MUA and (2) within an area defined as either EJA/MUA only or EJA & MUA. We decided to combine the two EJA/MUA neighborhood types to increase sample size and for ease of interpretation. To test for differential effects of risk factors across neighborhood type (i.e., moderation), we used the standardized *z*-test of differences between betas for independent samples (Paternoster, Brame, Mazerolle, & Piquero, 1998).

## Results

### GIS Spatial Analyses and Mapping

**Figure 1.**
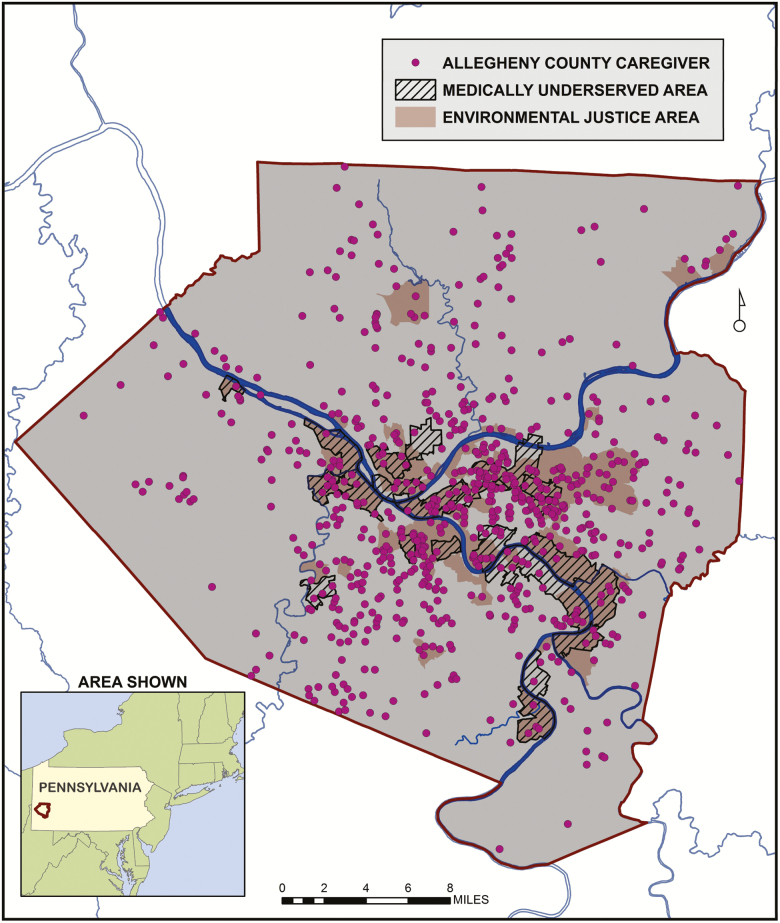
Caregiver cross-street locations are geocoded from the Pittsburgh Regional Caregiver Survey. Environmental Justice Areas are defined by the PADEP as any census tract where at least 20% of the population lives in poverty and/or 30% or more of the population is minority. Medically Underserved Area designations are based on the HRSA Index of Medical Underservice (IMU). IMU is calculated based on four criteria: the population to provider ratio, the percent of the population below the federal poverty level, the percent of the population over age 65, and the infant mortality rate.

**Figure 2.**
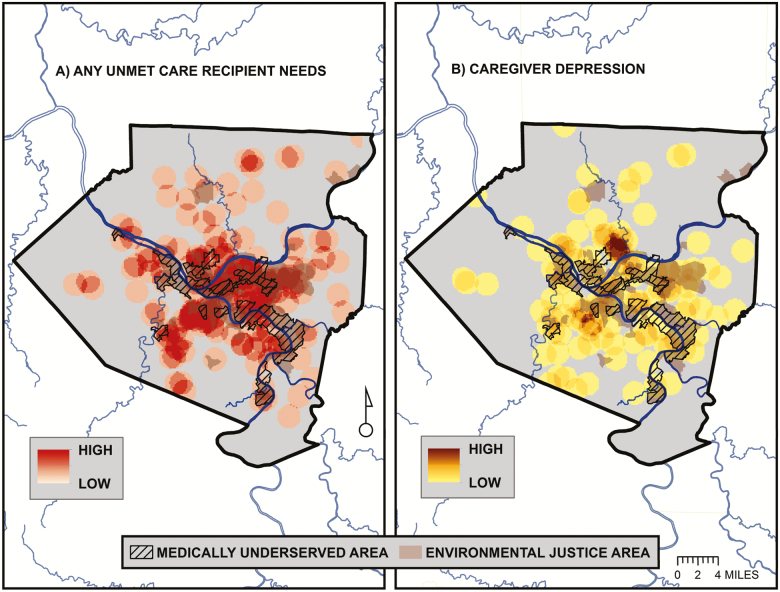
Point density maps of: (A) care recipients with unmet needs and (B) caregivers with depression based on caregiver responses to the Pittsburgh Regional Caregiver Survey. Point densities are calculated by summing the number of caregivers with unmet needs or depression within a defined neighborhood surrounding each point location and then dividing by the neighborhood area. Densities are then mapped on a continuous scale from high to low.

**Figure 3.**
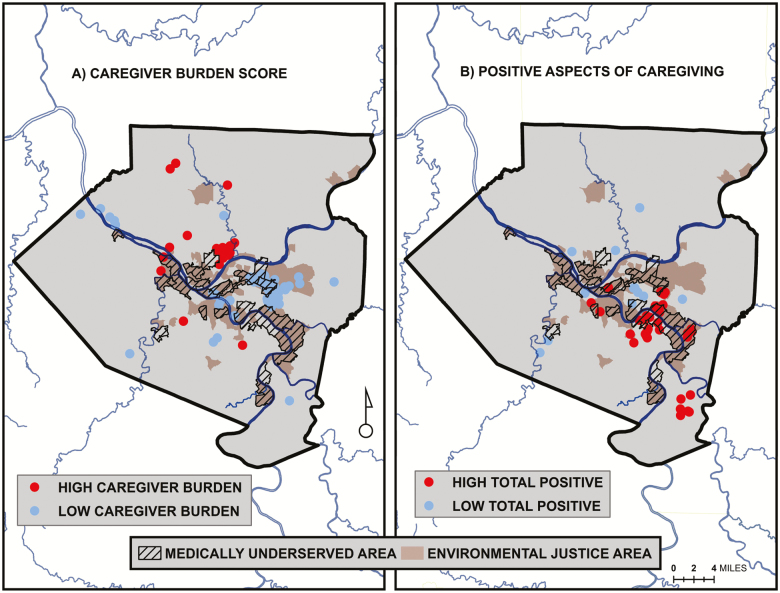
Cluster maps of summed continuous values for: (A) caregiver burden score and (B) positive aspects of caregiving from caregiver responses to the Pittsburgh Regional Caregiver Survey. Maps show the locations of statistically significant clusters of points with similarly high or low values identified using the Anselin Local Moran’s *I* statistic of spatial association.

The spatial distribution of caregiver locations and their relation to the EJA and MUA is shown in Figure 1. The map shows that, while clustered in the central urban core, caregivers are spatially distributed throughout the county. They are also located in non-EJAs/MUAs, EJAs/MUAs, and EJAs & MUAs. Point densities for the two binary survey responses are displayed as “heat maps” in Figure 2. High densities of caregivers with unmet care recipient need tend to be concentrated within EJA and MUA, while caregivers with depression tend to be more highly concentrated outside of these areas. Statistical clusters of the continuous caregiver burden and positive aspects of caregiving outcomes are shown in Figure 3. Clusters of points with high burden scores are seen outside of the EJA and MUA, while points with the highest positive aspects of caregiving scores are concentrated within them.

### Overall Sample Descriptive Statistics

As shown in Table 1, caregivers in the overall sample (*N* = 758) had an average age of 58.4 (*SD* = 12.1), while care recipients average age was 78.7 (*SD* = 12.2). The sample was 75.5% female, with 65% of care recipients also female. The sample included 18% African-American caregivers; over 57% had at least a bachelor’s degree; and nearly half reported an annual income of less than $50,000. The majority of the caregivers were the adult child of the care recipient; 42% co-resided with the recipient; while 32% of the recipients lived alone. Over half of the sample (50.7%) reported having two or more additional unpaid helpers for the care recipient. Slightly less than half the sample were currently employed; 17.9% had children under 18 living in their household; and 41.3% reported taking care of or assisting someone in addition to the care recipient. In terms of care recipient disability, about 55% were not AD patients and had two or fewer ADL needs; and 22% had three or more ADL needs, but no AD. About 12% of the care recipients had both AD and required help with three or more ADLs. More than 60% of the caregivers had been caring for the care recipient for at least 3 years, with 36.1% having been caregivers for more than 5 years. About two-thirds of the caregivers reported spending less than 20 hr per week helping the care recipient, while about 16% reported 40 or more hours per week.

### Sample Descriptive Statistics by Neighborhood

Table 1 shows that caregivers and care recipients living in EJA/MUA and urban neighborhoods tended to be younger. By definition, EJA/MUA/urban caregivers are more likely to be minority race, less educated, and have lower income. Caregivers from these less advantaged/underserved neighborhoods tended to be someone other than the care recipient’s spouse or adult child (e.g., a sibling or other family member, or friend). It is important to note that these differences are merely descriptive and do not imply that living in a particular neighborhood plays a causal role.

### Caregiver Risk Factor Descriptive Statistics: Overall and By Neighborhood

Table 2 shows that caregivers reported a mean suffering level for care recipients of 5.1 (*SD* = 2.3). Over half of the sample reported not having a choice in taking on caregiving. About 23% of the caregivers reported “fair” or “poor” self-rated health. Slightly less than half the sample responded “yes” to both social support items, while about 8% said “no” to both. Looking at neighborhood differences, fewer in the EJA, EJA/MUA, and urban neighborhoods reported not having a choice taking on the caregiving role. EJA and EJA/MUA caregivers were also more likely to report poor or fair health.

**Table 2.** Caregiver Risk Factor and Outcome Measures Descriptive Statistics for the Total Sample, by Neighborhood Type and Urban/Non-Urban Caregivers

		Neighborhood type			Urban/non-urban	
	Overall	Neither EJA nor MUA	EJA or MUA	EJA & MUA	Non-urban	Urban
	*N* = 758	*n* = 470	*n* = 159	*n* = 129	*n* = 388	*n* = 370
Caregiver risk factors						
Mean perceived CR suffering score	5.07	5.17	5.14	4.62	5.11	5.02
% With no choice in caregiving	54.5	57.9	52.8	44.2*	58.0	50.8*
% Reporting fair or poor health	23.2	19.8	30.2	27.1*	21.7	24.9
Caregiver social support						
% Low (score = 0)	7.8	7.0	6.9	11.6	7.2	8.4
% Medium (score = 1)	43.4	45.3	38.4	42.6	42.8	44.1
% High (score = 2)	48.8	47.7	54.7	45.7	50.0	47.6
Caregiver outcome measures						
% With CR who has unmet ADL/IADL/mobility needs	22.3	21.5	23.3	24.0	21.4	23.2
% Depressed (PHQ-2)	16.1	18.1	15.1	10.1	16.5	15.7
Mean caregiving burden score	2.06	2.26	1.98	1.45**	2.19	1.92*
Mean positive aspects of caregiving score	5.49	5.26	5.72	6.06**	5.42	5.57

*Note*: ADL = activities of daily living; CR = care recipient; EJA = Environmental Justice Area; IADL = instrumental activities of daily living; MUA = Medically Underserved Area; PHQ = Patient Health Questionnaire.

**p ≤ .01; *p ≤ .05 for neighborhood type; urban/non-urban in ANOVA (CR suffering, caregiver burden, positive aspects of caregiving) or χ ^2^ test.

### Caregiver Outcomes Descriptive Statistics: Overall and by Neighborhood

About 22% of the care recipients’ had at least one unmet ADL/IADL/mobility need for assistance. In addition, 16% of the caregivers met the PHQ-2 cutoff criteria for depression. On average, caregivers scored a mean of 2.1 (*SD* = 1.9) on the 7-point burden index, while averaging 5.5 (*SD* = 2.1) on the 8-point positive aspects of caregiving scale. Looking at differences by neighborhood, the bivariate findings mirror the GIS mapping results presented in Figures 2 and 3. Caregivers living in EJA & MUA neighborhoods scored significantly lower on the caregiver burden index, and scored significantly higher on the positive aspects of caregiving scale. Caregivers in the EJA/MUA neighborhoods scored at intermediate levels on all three outcomes, and do not differ significantly from the EJA & MUA group. Differences between the EJA-only caregivers and the neither EJA nor MUA group are not significant using the Scheffe post hoc test.

### Multivariable Caregiver Outcome Models Testing for Neighborhood Main Effects

Table 3 shows results of the multivariable logistic regression models for the binary outcomes (recipient unmet needs for care; meeting the cutoff for depression). Table 4 shows results of the negative binomial models for the caregiver burden index, and the OLS models for the positive aspects of caregiving scale.

**Table 3.** Logistic Regression Risk Factor Models for Unmet Care Recipient Needs and Caregiver Depression, With and Without Objective Neighborhood Characteristics (*N* = 745)

	Unmet CR needs		CG depression (PHQ-2)	
	Model 1	Model 2	Model 1	Model 2
Sociodemographic				
CG age	1.02 (.095)^+^	1.02 (.074)^+^	0.99 (.428)	0.99 (.372)
CR Age	0.98 (.054)^+^	0.98 (.059)^+^	1.00 (.999)	1.00 (.937)
Female CG	1.31 (.258)	1.32 (.248)	1.11 (.731)	1.03 (.918)
Female CR	0.92 (.703)	0.91 (.683)	0.86 (.598)	0.87 (.624)
African-American	1.11 (.680)	0.98 (.948)	0.71 (.296)	1.05 (.892)
Some college	0.60 (.058)^+^	0.60 (.053)^+^	0.88 (.708)	0.86 (.659)
Bachelor’s degree	0.68 (.185)	0.69 (.193)	2.02 (.039)*	1.92 (.057)^+^
Master’s degree or higher	0.83 (.540)	0.83 (.536)	0.65 (.305)	0.62 (.249)
≤$20,000	1.05 (.874)	1.04 (.908)	1.28 (.466)	1.22 (.571)
>$50,000	1.17 (.519)	1.19 (.481)	0.53 (.033)*	0.51 (.022)*
Caregiving context				
Adult child CG	0.71 (.156)	0.72 (.180)	1.26 (.470)	1.22 (.536)
Spouse CG	0.36 (.004)**	0.36 (.005)**	0.86 (.729)	0.86 (.728)
CG–CR co-reside	1.58 (.125)	1.60 (.116)	1.79 (.104)	1.77 (.114)
CR lives alone	1.77 (.032)*	1.78 (.031)*	1.09 (.811)	1.12 (.742)
# Other CGs	0.93 (.224)	0.92 (.206)	0.95 (.485)	0.96 (.605)
Employed	1.16 (.503)	1.16 (.498)	0.97 (.907)	0.98 (.944)
Children in HH	1.32 (.327)	1.35 (.289)	1.01 (.986)	0.97 (.934)
Caring for other than CR	1.02 (.933)	1.02 (.928)	1.23 (.401)	1.22 (.429)
Care recipient disability				
CR AD only	1.69 (.065)^+^	1.71 (.059)^+^	1.11 (.794)	1.08 (.853)
CR three or more ADLs only	0.80 (.403)	0.79 (.383)	2.05 (.017)*	2.08 (.016)*
CR AD and three or more ADLs	0.88 (.699)	0.88 (.704)	1.66 (.180)	1.64 (.194)
Caregiving intensity				
Hours per week spent CG	1.10 (.365)	1.10 (.345)	1.13 (.328)	1.12 (.371)
Duration of CG	0.94 (.443)	0.94 (.431)	0.99 (.932)	1.00 (.983)
Additional risk factors				
Perceived CR suffering	1.13 (.005)**	1.13 (.005)**	1.10 (.066)^+^	1.09 (.086)^+^
No choice in caregiving	1.07 (.728)	1.08 (.687)	2.30 (.001)**	2.22 (.002)**
Fair or poor CG health	1.07 (.763)	1.06 (.809)	2.88 (<.001)**	3.01 (<.001)**
CG social support	0.64 (.005)**	0.64 (.005)**	0.47 (<.001)**	0.45 (<.001)**
Neighborhood factors				
EJA/MUA neighborhood		1.25 (.376)		0.66 (.191)
EJA & MUA neighborhood		1.22 (.542)		0.41 (.036)*
Urban neighborhood		1.06 (.775)		1.14 (.617)
Model *R*^2^	.113	.115	.269	.278

*Note*: AD = Alzheimer’s disease; ADL = activities of daily living; CG = caregiver; CR = care recipient; EJA = Environmental Justice Area; HH = household; MUA = Medically Underserved Area; PHQ = Patient Health Questionnaire. Table entries are odds ratios (ORs) and (*p* values).

**p ≤ .01; *p ≤ .05; ^+^*p* ≤ .10.

**Table 4.** Negative Binomial Model for Caregiver Burden and Ordinary Least Squares Model for Positive Aspects of Caregiving, With and Without Objective Neighborhood Characteristics

	CG burden (*N* = 745)		Positive aspects of caregiving (*N* = 729)	
	Model 1	Model 2	Model 1	Model 2
Sociodemographic				
CG age	−.003 (.635)	−.003 (.592)	−.001 (.889)	.000 (.964)
CR age	.003 (.580)	.003 (.574)	.013 (.093)^+^	.014 (.068)^+^
Female CG	.339 (.005)**	.331 (.006)**	.154 (.389)	.172 (.336)
Female CR	−.152 (.180)	−.150 (.187)	.137 (.432)	.122 (.483)
African-American	−.321 (.021)*	−.273 (.090)^+^	.644 (.002)**	.440 (.065)^+^
Some college	.069 (.624)	.068 (.633)	−.287 (.180)	−.287 (.181)
Bachelor’s degree	.168 (.268)	.165 (.275)	−.377 (.104)	−.366 (.115)
Master’s degree or higher	.275 (.085)^+^	.276 (.086)^+^	−.821 (.001)**	−.800 (.001)**
≤$20,000	−.029 (.858)	−.030 (.852)	−.177 (.474)	−.159 (.520)
>$50,000	.036 (.775)	.027 (.827)	−.431 (.026)*	−.408 (.037)*
Caregiving context				
Adult child CG	−.019 (.879)	−.027 (.829)	−.095 (.619)	−.080 (.676)
Spouse CG	−.263 (.158)	−.263 (.159)	.576 (.051)^+^	.582 (.049)*
CG−CR co-reside	.277 (.060)^+^	.272 (.065)^+^	−.225 (.321)	−.211 (.353)
CR lives alone	.070 (.594)	.069 (.597)	−.280 (.159)	−.279 (.158)
# Other CGs	−.008 (.805)	−.007 (.825)	−.019 (.691)	−.025 (.612)
Employed	.095 (.396)	.095 (.392)	.270 (.116)	.271 (.115)
Children in HH	.188 (.170)	.180 (.190)	−.258 (.239)	−.222 (.312)
Caring for other than CR	.119 (.246)	.119 (.246)	−.127 (.420)	−.117 (.457)
Care recipient disability				
CR AD only	.215 (.168)	.211 (.176)	.049 (.841)	.052 (.831)
CR three or more ADLs only	.291 (.023)*	.293 (.022)*	.039 (.849)	.017 (.934)
CR AD and three or more ADLs	.451 (.004)**	.449 (.004)**	.046 (.856)	.051 (.838)
Caregiving intensity				
Hours per week spent CG	.159 (.002)**	.157 (.003)**	.240 (.003)**	.243 (.003)
Duration of CG	.044 (.288)	.043 (.302)	.167 (.008)**	.160 (.011)*
Additional risk factors				
Perceived CR suffering	.114 (<.001)**	.113 (<.001)**	−.026 (.429)	−.024 (.470)
No choice in caregiving	.456 (<.001)**	.452 (<.001)**	−.702 (<.001)**	−.690 (<.001)**
Fair or poor CG health	.407 (<.001)**	.408 (<.001)**	−.269 (.141)	−.294 (.108)
CG social support	−.117 (.151)	−.120 (.142)	.187 (.145)	.195 (.131)
Neighborhood factors				
EJA/MUA neighborhood		−.040 (.759)		.367 (.069)^+^
EJA & MUA neighborhood		−.090 (.598)		.496 (.056)^+^
Urban neighborhood		−.005 (.967)		−.171 (.303)
Model fit (log likelihood) for CG burden; model *R*^2^ for positive aspects of CG	−1353.83	−1353.66	.145	.151

*Note*: AD = Alzheimer’s disease; ADL = activities of daily living; CG = caregiver; CR = care recipient; EJA = Environmental Justice Area; HH = household; MUA = Medically Underserved Area. Table entries are unstandardized betas and (*p* values).

**p ≤ .01; *p ≤ .05; ^+^*p* ≤ .10.

#### Care recipient unmet needs

As shown in model 1 of Table 3 (left panel; *R*^2^ = .113), those with care recipients who live alone (*B* = 0.571, exp(*B*) = 1.77, 95% CI exp(*B*) [1.05, 2.98]), those who perceive greater care recipient suffering (*B* = 0.118, exp(*B*) = 1.13, 95% CI exp(*B*) [1.04, 1.22]), and those with lower social support (*B* = −0.454, exp(*B*) = 0.64, 95% CI exp(*B*) [0.46, 0.87]) were more likely to report unmet recipient needs for care. In addition, spouse caregivers (*B* = −1.02, exp(*B*) = 0.36, 95% CI exp(*B*) [0.18, 0.73]) were less likely to report unmet needs. In the second model, neighborhood factors were not significantly related to care recipient unmet needs after adjusting for the individual-level risk factors and the significant predictors reported above were unchanged.

#### Caregiver depression

The individual-level model for caregiver depression in Table 3 (model 1, right panel; *R*^2^ = .269) shows that caregivers with a Bachelor’s degree (*B* = 0.703, exp(*B*) = 2.02, 95% CI exp(*B*) [1.04, 3.93]), caregivers of recipients with three or more ADL needs (*B* = 0.720, exp(*B*) = 2.05, 95% CI exp(*B*) [1.14, 3.71]), those who did not have a choice in becoming a caregiver (*B* = 0.832, exp(*B*) = 2.30, 95% CI exp(*B*) [1.41, 3.76]), caregivers in poor or fair health (*B* = 1.059, exp(*B*) = 2.88, 95% CI exp(*B*) [1.78, 4.69]), and those with lower social support (*B* = −0.757, exp(*B*) = 0.47, 95% CI exp(*B*) [0.32, 0.68] were more likely to be depressed. Higher-income caregivers making $50,000 or more (*B* = −0.633, exp(*B*) = 0.53, 95% CI exp(*B*) [0.30, 0.95]) were less likely to be depressed. In the second model, caregivers who live in neighborhoods designated as both EJA and MUA (*B* = −0.900, exp(*B*) = 0.41, 95% CI exp(*B*) [0.18, 0.95]) were less likely to be depressed.

#### Caregiver burden

Table 4 (model 1, left panel) shows that there were several significant individual-level predictors of caregiver burden. Female caregivers (*B* = 0.339 [*SE* = 0.119]), when the recipient has three or more ADL needs (*B* = 0.291 [*SE* = 0.128]), or three or more ADL needs plus AD (*B* = 0.451 [*SE* = 0.157]), and caregivers who report spending more hours per week providing care (*B* = 0.159 [*SE* = 0.052]) have significantly higher burden scores. African-American caregivers (*B* = −0.321 [*SE* = 0.138]) report lower burden scores. In addition, those who perceived more recipient suffering (*B* = 0.114 [*SE* = 0.022]), those who did not have a choice in becoming a caregiver (*B* = 0.456 [*SE* = 0.100]), and caregivers in poor or fair health (*B* = 0.407 [*SE* = 0.115]) had higher burden scores. In the second model, neighborhood factors were not significantly related to caregiver burden after adjusting for the individual-level risk factors.

#### Positive aspects of caregiving

The individual-level model for positive aspects of caregiving (Table 4, model 1, right panel; *R*^2^ = .145) shows that African-American caregivers (*B* = 0.644 [*SE* = 0.206]), those who had been caring for the care recipient for a longer duration (*B* = 0.167 [*SE* = 0.03]), and those who spend more hours per week caregiving (*B* = 0.240 [*SE* = 0.081]) had higher positive aspects of caregiving scores. Caregivers with a Master’s degree or higher (*B* = −0.821 [*SE* = 0.244]), those earning more than $50,000 per year (*B* = −0.431 [*SE* = 0.193]), and those who felt they had no choice in becoming a caregiver (*B* = −0.702 [*SE* = 0.154]) had lower positive aspects of caregiving scores. In the second model, there were trends (*p* < .10) for caregivers who live in neighborhoods designated as either EJA or MUA (*B* = 0.367 [*SE* = 0.201]); and both EJAs and MUAs (*B* = 0.496 [*SE* = 0.259]) have higher positive aspects of caregiving scores.

### Supplemental Caregiver Outcome Models Testing for Neighborhood Moderator Effects

Supplementary Tables 1 and 2 summarize the models used to test for neighborhood moderator effects. Although the tables show beta differences for effects at the *p* <.10 level, we only discuss those reaching traditional (*p* < .05) significance levels. Caregivers with Masters degrees or higher were less likely to report unmet care recipient needs, while those who were employed, had children in the household, and perceived more care recipient suffering were more likely to report unmet care recipient needs, but only among EJA/MUA caregivers. Younger caregivers from EJA/MUA neighborhoods, but not those from other neighborhoods, were more likely to meet caregiver depression cutoff scores. In the models for caregiver burden, caregivers of older care recipients were more burdened, but only within EJA/MUA neighborhoods. Adult child caregivers were more likely to report positive aspects of caregiving within EJA and MUA neighborhoods; while non-EJA/MUA adult children reported fewer positive aspects of caregiving. Lastly, caregivers whose care recipient lives alone were less likely to report positive aspects of caregiving, but only within non-EJA/MUA neighborhoods.

## Discussion

This paper combined GIS and survey methods to examine the impact of caregiver neighborhood on caregiver outcomes. The key findings were that caregiver depression and burden tended to be spatially concentrated outside of relatively disadvantaged/underserved neighborhoods, while positive aspects of caregiving were concentrated within disadvantaged/underserved areas. These effects were less robust in multivariable statistical models adjusting for a large number of individual-level risk factors, suggesting some but not complete covariation of locational variables with individual differences. Our findings were most robust for the caregiver depression outcome, which was lower for those living in areas defined as both EJA & MUA. Those living in areas designated as EJA/MUA only were not significantly less depressed. Although our findings for positive aspects of caregiving (i.e., caregiving gains) did not meet traditional significance levels in multivariate adjusted models, both the EJA & MUA and EJA/MUA groups revealed trends (*p* < .10) showing more positive aspects. Unadjusted bivariate results showed significantly more positive aspects of caregiving in relatively disadvantaged/underserved neighborhood areas.

Our findings suggest that locational effects may be an important new arena for exploring caregiver and care recipient outcomes. In particular, they suggest that caregivers living in the most disadvantaged/underserved areas were less likely to be depressed, and tended to report more positive aspects of caregiving. These findings are surprising and suggest that socioeconomic disadvantage does not necessarily translate into poor caregiver outcomes. Findings also suggest that neighborhood characteristics play a moderating role on the impact of individual-level risk factors on caregiver outcomes and care recipient outcomes.

Future research should replicate and extend these findings as well as explore underlying mechanisms that might explain these counterintuitive effects. It is possible that caregivers from disadvantaged areas benefit from coping mechanisms that have developed over time by dealing with multiple stressors, which make caregiving relatively less challenging. These caregivers may also simply have more expectation that they will provide care to loved ones including aging parents, and thus take on the role more willingly and derive more benefits. The finding that adult children from disadvantaged/underserved neighborhoods reported more positive aspects of caregiving while adult children from more advantaged areas reported fewer positive aspects is consistent with this interpretation. There could also be social comparison effects operating, in which caregivers in more disadvantaged areas are deriving psychological benefits by comparing themselves to others who are even worse off (Stewart, Chipperfield, Ruthig, & Heckhausen, 2013).

It is important to note that while this study focused on outcomes typically examined in caregiver research, they may not be ideal for detecting neighborhood effects. Much of the literature on neighborhood effects focuses on physical health outcomes or proxies for physical health effects, such as exposure to air pollution (Hajat et al., 2015; Hill et al., 2019), unequal access to healthy food (Hilmers et al., 2012), lower cancer screening rates (Wong, 2015), increased cardiovascular risk factors (Allen et al., 2011), and preventable hospitalizations (Parchman & Culler, 1999). Thus, future research in this area may benefit from expanding the range of outcomes to include physical health of the caregiver and institutionalization, health care utilization (e.g., emergency room visits), and mortality of the care recipient. However, this study makes a contribution to the neighborhood effects literature by illuminating potential psychological effects of the neighborhood environment for caregivers of older adults.

The paper demonstrates the value of using GIS methods to study caregiving. Caregivers living in EJA/MUA areas have different individual characteristics (age, sex, relationship to care recipient), risks factors (support, poor health), and outcomes (depression, positive aspects of caregiving) compared to those living outside of those areas. We found distinct spatial patterning of caregiver survey results across Allegheny County, PA, and GIS analyses and mapping enhance the value of traditional caregiver survey responses. Geospatial techniques allowed us to show that caregivers exhibit differential clustering in terms of both negative and positive aspects of caregiving, each with a different pattern of association with neighborhood-level socioeconomic characteristics. These preliminary findings may help in targeting intervention in places where neighborhood characteristics could increase the need for caregiver assistance, but further work is needed to investigate the proper scale at which the effect of neighborhood environment on caregiver health may operate.

In addition, while we found no direct neighborhood-level effects on unmet care recipient needs, supplemental analyses showed that neighborhood type moderated the effects of individual-level risk factors on unmet needs. For care recipients living in EJA/MUA neighborhoods only, having a caregiver with higher education (at least some college) was related to fewer unmet needs. Also, in EJA/MUA neighborhoods, employed caregivers and those who reported children under 18 in the household were more likely to have care recipients with unmet needs, which was not found in the non-EJA/MUA areas. Neighborhood environment also moderated the effects of select individual-level risk factors on caregiver depression, caregiver burden, and positive aspects of caregiving. These analyses reveal the complexities of neighborhood effects on caregiving, and the moderating role that environmental context might play in caregiver and care recipient outcomes.

This study has certain limitations that should be noted. The use of a cross-sectional design limits the ability to make causal statements, although this is less of an issue when examining neighborhood contextual effects. We attempted to adjust for neighborhood selection effects by including a variety of individual-level sociodemographic and caregiving context variables, but we cannot be sure that some of our findings are not simply the result where caregivers have chosen to live. Also, as noted above, three of our outcome measures are subjective/psychological, and we did not examine physical health effects, where neighborhood may have the largest impact. Spatial analyses are sensitive to scale and the EJAs and MUAs used to delineate underprivileged neighborhoods may be defined too coarsely to effectively model individual caregiver environment interactions. Lastly, our study was conducted in a single predominantly urban county in Pennsylvania (Pittsburgh), and thus our findings might not generalize to other locations.

## Conclusions and Implications

This study combined GIS and survey methods to study the impact of living in relatively disadvantaged/underserved neighborhoods on caregiver outcomes. We report somewhat surprising results showing that caregivers in living in these potentially challenging environments were less depressed and tended to report more gains from caregiving after adjusting for known risk factors. Results suggest that socioeconomic disadvantage does not necessarily translate into poor caregiver outcomes. Understanding the mechanism for these effects is important to designing effective caregiver interventions. Interventions for caregivers in disadvantaged areas could build upon psychological strengths and resources, while those for caregivers in other areas should focus on enhancing mental health. The paper also demonstrates the value of using GIS methods to study caregiving. GIS analyses and mapping enhance the analytic value of traditional caregiver survey responses.

## Supplementary Material

igz025_suppl_Supplementary_Table
