# Supplementary material for: Caregiving and Place: Combining Geographic Information System (GIS) and Survey Methods to Examine Neighborhood Context and Caregiver Outcomes
Source: Innov Aging. 2019 Aug 23;3(3):igz025. doi: 10.1093/geroni/igz025 (PMC6735773; doi:10.1093/geroni/igz025)
Supplement: igz025_suppl_Supplementary_Table [file igz025_suppl_supplementary_table.docx]

Supplemental Table 1. Logistic regression risk factor models for unmet care recipient needs and caregiver depression, estimated separately within non-EJ / MUA and EJ / MUA / EJ & MUA neighborhoods.

|  | **Any Unmet CR Needs** | | **CG Depression (PHQ-2)** | |
| --- | --- | --- | --- | --- |
|  | **Neither EJA nor MUA**  **(n = 463)** | **EJA / MUA /**  **EJA & MUA**  **(n = 282)** | **Neither EJA nor MUA**  **(n = 463)** | **EJA / MUA /**  **EJA & MUA**  **(n = 282)** |
| ***Socio-demographic*** |  |  |  |  |
| CG Age | 1.01 (.666) | 1.04 (.033)* | 1.01 (.728) | 0.95 (.027)* |
| CR Age | 0.97 (.035)* | 1.00 (.950) | 0.98 (.158) | 1.04 (.147) |
| Female CG | 1.06 (.863) | 1.29 (.529) | 1.19 (.635) | 0.85 (.781) |
| Female CR | 1.17 (.622) | 0.88 (.738) | 0.78 (.467) | 1.09 (.885) |
| African-American | 0.51 (.414) | 1.17 (.659) | 1.50 (.567) | 0.94 (.913) |
| Some college | 0.88 (.752) | 0.34 (.010)* | 0.92 (.839) | 0.96 (.946) |
| Bachelor's degree | 0.89 (.764) | 0.37 (.041)* | 1.58 (.289) | 5.48 (.012)* |
| Master's degree or higher | 1.26 (.559) | 0.29 (.035)* | 0.65 (.388) | 0.97 (.973) |
| $20,000 or less | 0.71 (.471) | 1.48 (.421) | 1.27 (.602) | 1.36 (.624) |
| > $50,000 | 1.15 (.678) | 1.57 (.303) | 0.52 (.071)+ | 0.37 (.122) |
| ***Caregiving context*** |  |  |  |  |
| Adult child CG | 0.72 (.376) | 0.79 (.528) | 1.62 (.290) | 1.49 (.492) |
| Spouse CG | 0.32 (.018)* | 0.51 (.291) | 0.53 (.236) | 5.06 (.092)+ |
| CG-CR co-reside | 2.14 (.076)+ | 1.23 (.670) | 2.22 (.088)+ | 1.00 (.996) |
| CR lives alone | 2.83 (.008)** | 1.22 (.651) | 1.82 (.189) | 0.46 (.232) |
| # Other CGs | 0.91 (.300) | 0.93 (.502) | 0.93 (.510) | 0.84 (.275) |
| Employed | 0.62 (.113) | 3.53 (.001)** | 1.05 (.898) | 0.87 (.794) |
| Children in HH | 0.95 (.906) | 3.37 (.008)** | 1.08 (.859) | 0.65 (.546) |
| Caring for Other than CR | 0.90 (.708) | 1.00 (.999) | 1.16 (.631) | 1.41 (.488) |
| ***Care recipient disability*** |  |  |  |  |
| CR Alzheimer's (AD) only | 2.27 (.023)* | 0.84 (.761) | 1.12 (.813) | 1.53 (.651) |
| CR 3 or more ADLs only | 0.70 (.365) | 0.94 (.874) | 1.70 (.174) | 4.41 (.009)** |
| CR AD and 3 or more ADLs | 0.82 (.678) | 0.93 (.896) | 1.98 (.140) | 1.58 (.581) |
| ***Caregiving intensity*** |  |  |  |  |
| Hours per Week spent CG | 1.17 (.288) | 0.89 (.473) | 1.27 (.139) | 0.94 (.810) |
| Duration of CG | 0.99 (.950) | 0.88 (.318) | 0.94 (.630) | 1.17 (.463) |
| ***Additional risk factors*** |  |  |  |  |
| Perceived CR suffering | 1.09 (.166) | 1.34 (<.001)** | 1.10 (.151) | 1.03 (.793) |
| No choice in caregiving | 1.29 (.361) | 1.00 (.995) | 1.84 (.062)+ | 2.55 (.056)+ |
| Fair or poor CG health | 1.14 (.688) | 0.80 (.543) | 3.04 (.001)** | 4.39 (.003)** |
| CG social support | 0.50 (.001)** | 0.97 (.928) | 0.56 (.019)* | 0.33 (.003)** |
| ***Neighborhood factors***  Urban neighborhood | 1.00 (.996) | 1.28 (.538) | 0.84 (.580) | 2.07 (.219) |
| **Model R^2^** | .193 | .216 | .296 | .371 |

Table entries are Odds ratios (OR) and (p-values); ** p < .01; * p < .05; + p < .10

Darkly shaded cells indicate variables for which the effects differ at p < .05 by neighborhood type using z-test for differences between betas. Lightly shaded cell effects differ at p < .10 by neighborhood type.

Supplemental Table 2. Negative binomial model for caregiver burden and OLS regression models for positive aspects of caregiving, estimated separately within non-EJ / MUA and EJ / MUA / EJ & MUA neighborhoods.

|  | **CG Burden** | | **Positive Aspects of Caregiving** | |
| --- | --- | --- | --- | --- |
|  | **Neither EJA nor MUA**  **(n = 463)** | **EJA / MUA /**  **EJA & MUA**  **(n = 282)** | **Neither EJA nor MUA**  **(n = 454)** | **EJA / MUA /**  **EJA & MUA**  **(n = 275)** |
| ***Socio-demographic***  CG Age | .000 (.974) | -.008 (.337) | -.005 (.710) | .002 (.863) |
| CR Age | -.006 (.401) | .016 (.062)+ | .025 (.021)* | .011 (.357) |
| Female CG | .347 (.026)* | .285 (.163) | .131 (.577) | .232 (.419) |
| Female CR | -.148 (.315) | -.168 (.399) | .108 (.633) | .094 (.743) |
| African-American | -.381 (.257) | -.114 (.549) | .822 (.101) | .313 (.248) |
| Some college | .150 (.435) | -.075 (.734) | -.386 (.194) | -.134 (.674) |
| Bachelor's degree | .006 (.976) | .319 (.213) | -.358 (.240) | -.426 (.263) |
| Master's degree or higher | .178 (.381) | .413 (.153) | -.679 (.030) | -1.20 (.006)** |
| $20,000 or less | -.088 (.698) | -.088 (.728) | -.278 (.457) | -.185 (.601) |
| > $50,000 | .019 (.908) | .034 (.879) | -.388 (.131) | -.360 (.273) |
| ***Caregiving context*** |  |  |  |  |
| Adult child CG | .090 (.603) | -.074 (.704) | -.487 (.076)+ | .313 (.264) |
| Spouse CG | -.395 (.102) | .063 (.849) | .718 (.069)+ | .013 (.979) |
| CG-CR co-reside | .212 (.264) | .414 (.110) | -.409 (.180) | .218 (.546) |
| CR lives alone | .027 (.872) | .222 (.324) | -.678 (.010)** | .183 (.572) |
| # Other CGs | -.008 (.851) | -.022 (.691) | .000 (.998) | -.048 (.549) |
| Employed | .120 (.404) | .060 (.754) | .282 (.223) | .212 (.435) |
| Children in HH | .305 (.099)+ | .025 (.915) | -.180 (.555) | -.134 (.699) |
| Caring for Other than CR | .069 (.602) | .265 (.140) | -.215 (.302) | .022 (.930) |
| ***Care recipient disability*** |  |  |  |  |
| CR Alzheimer's (AD) only | .233 (.216) | .202 (.503) | .016 (.959) | -.099 (.818) |
| CR 3 or more ADLs only | .251 (.140) | .354 (.088) | .120 (.668) | -.116 (.704) |
| CR AD and 3 or more ADLs | .416 (.035)* | .527 (.062)+ | -.170 (.601) | .323 (.454) |
| ***Caregiving intensity*** |  |  |  |  |
| Hours per Week spent CG | .223 (.002)** | .054 (.521) | .190 (.091)+ | .253 (.041)* |
| Duration of CG | .099 (.064)+ | -.069 (.329) | .102 (.215) | .207 (.044)* |
| ***Additional risk factors*** |  |  |  |  |
| Perceived CR suffering | .109 (<.001)** | .119 (.002)** | -.070 (.119) | .044 (.415) |
| No choice in caregiving | .462 (<.001)** | .441 (.010)** | -.633 (.002)** | -.671 (.006)** |
| Fair or poor CG health | .538 (.001)** | .233 (.235) | -.015 (.954) | -.564 (.045) |
| CG social support | -.126 (.239) | -.073 (.604) | .212 (.221) | .149 (.465) |
| ***Neighborhood factors*** |  |  |  |  |
| Urban neighborhood | -.040 (.765) | .062 (.757) | -.126 (.544) | -.117 (.685) |
| **Model fit (Log Likelihood) for CG burden; Model R^2^  for positive aspects of CG** | -888.76 | -474.98 | .147 | .194 |

Table entries are unstandardized betas and (p-values). ** p < .01; * p < .05; + p < .10

Darkly shaded cells indicate variables for which the effects differ at p < .05 by neighborhood type using z-test for differences between betas. Lightly shaded cell effects differ at p < .10 by neighborhood type.
